# Torsion and Rupture of a Wandering Pelvic Accessory Spleen Associated with Essential Thrombocythemia: A Case Report

**DOI:** 10.70352/scrj.cr.26-0232

**Published:** 2026-08-22

**Authors:** Aoi Suzuki, Taku Sato, Fumi Hasegawa, Megumu Enjoji

**Affiliations:** 1 Department of Surgery, Japan Agricultural Co-operatives Toride Medical Center, Toride, Ibaraki, Japan; 2 Department of Surgery, Japanese Red Cross Musashino Hospital, Musashino, Tokyo, Japan

**Keywords:** pelvic accessory spleen, wandering accessory spleen, essential thrombocythemia

## Abstract

**INTRODUCTION:**

The pelvic accessory spleen is a rare anomaly, and its diagnosis is challenging. Essential thrombocythemia (ET) is a myeloproliferative neoplasm that can cause secondary myelofibrosis and splenomegaly. We report a case of torsion and rupture of a large wandering pelvic accessory spleen associated with ET.

**CASE PRESENTATION:**

A 62-year-old man with a history of ET presented with acute abdominal pain. Contrast-enhanced CT revealed a 9-cm pelvic mass with surrounding high-density fluid consistent with a hematoma, indicating its rupture. He was diagnosed with ET 8 years earlier, and disease control had gradually worsened. Splenomegaly and an 8-cm pelvic mass were detected 2 months before onset using CT and MRI. The signal-intensity pattern of the pelvic mass and the spleen on MRI was identical. Therefore, the mass was considered an accessory spleen rather than a tumor. Emergency laparotomy was performed. The pelvic mass was a wandering accessory spleen. There was no supportive tissue, except for a cord-like vascular pedicle. The mass was easily resected by ligating the pedicle. Splenomegaly was observed in the left lateral abdomen. In this case, both the native and pelvic accessory spleens were enlarged because of the progression of ET. The patient was discharged on POD 16.

**CONCLUSIONS:**

Awareness of pelvic accessory spleen and its torsion is important in the differential diagnosis of pelvic masses and acute abdominal pain. When a large pelvic accessory spleen is identified, elective resection should be considered to prevent torsion or rupture. Although secondary enlargement of an accessory spleen after splenectomy has been reported, simultaneous enlargement of both the native and accessory spleens is exceptionally rare. To our knowledge, this is the first report describing the pathophysiology of simultaneous enlargement of the native and accessory spleens in association with a hematological disease.

## Abbreviations

ADC
: apparent diffusion coefficient
AIHA
: autoimmune hemolytic anemia
DWI
: diffusion-weighted imaging
ET
: essential thrombocythemia
GIST
: gastrointestinal stromal tumor
HCV
: hepatitis C virus
HDRBC
: heat-damaged red blood cell
ITP
: immune thrombocytopenic purpura
JAK2
: Janus kinase 2
SC
: sulfur colloid
Tc-99m
: Technetium-99m
US
: ultrasonography

## INTRODUCTION

Accessory spleens are ectopic splenic tissues identified in approximately 10%–30% of autopsy cases.^1)^ Clinically, accessory spleens are often detected incidentally on radiographic imaging studies, including US and CT.^2,3)^ Most accessory spleens were asymptomatic and <4-cm in diameter.^3)^ The risk of torsion is high in patients with accessory spleens >6-cm; therefore, prophylactic surgical resection of large accessory spleens is recommended.^4)^

An accessory spleen is typically located in the left upper abdomen. In approximately 75% of cases, the accessory spleen is located at the splenic hilum, and in approximately 20% of cases, it is located at the pancreatic tail. Furthermore, it is found in the greater omentum, the mesocolon, and the pancreas. Reportedly its locations include the retroperitoneum, the mediastinum, the pelvis, and the scrotum.^5)^ Pelvic accessory spleens have been reported in only 18 cases, including this one.^6–22)^

Feeding vessels for pelvic accessory spleens arise from gastroepiploic vessels or splenic hilum arterial branches.^18)^ In some cases, an elongation of the vascular pedicle and a lack of suspensory ligaments are observed, resulting in excessive mobility. These accessory spleens are “wandering accessory spleens.”^4,5)^ The risk of torsion is high in pelvic wandering spleens.^14)^

ET is a myeloproliferative neoplasm characterized by clonal hematopoietic stem cell abnormalities, marked thrombocytosis, megakaryocytic hyperplasia, and symptoms including bleeding and thrombosis.^23)^ The diagnostic criteria include a platelet count >450 × 10^9^/L. Generally, its prognosis is favorable^24)^; however, approximately 12% of cases progress to secondary myelofibrosis.^23)^ Extramedullary hematopoiesis frequently occurs in the spleen, leading to splenomegaly.^25,26)^ To the best of our knowledge, this is the first case report describing the pathophysiology of simultaneous enlargement of an accessory spleen and splenomegaly associated with ET.

## CASE PRESENTATION

**Fig. 1:**
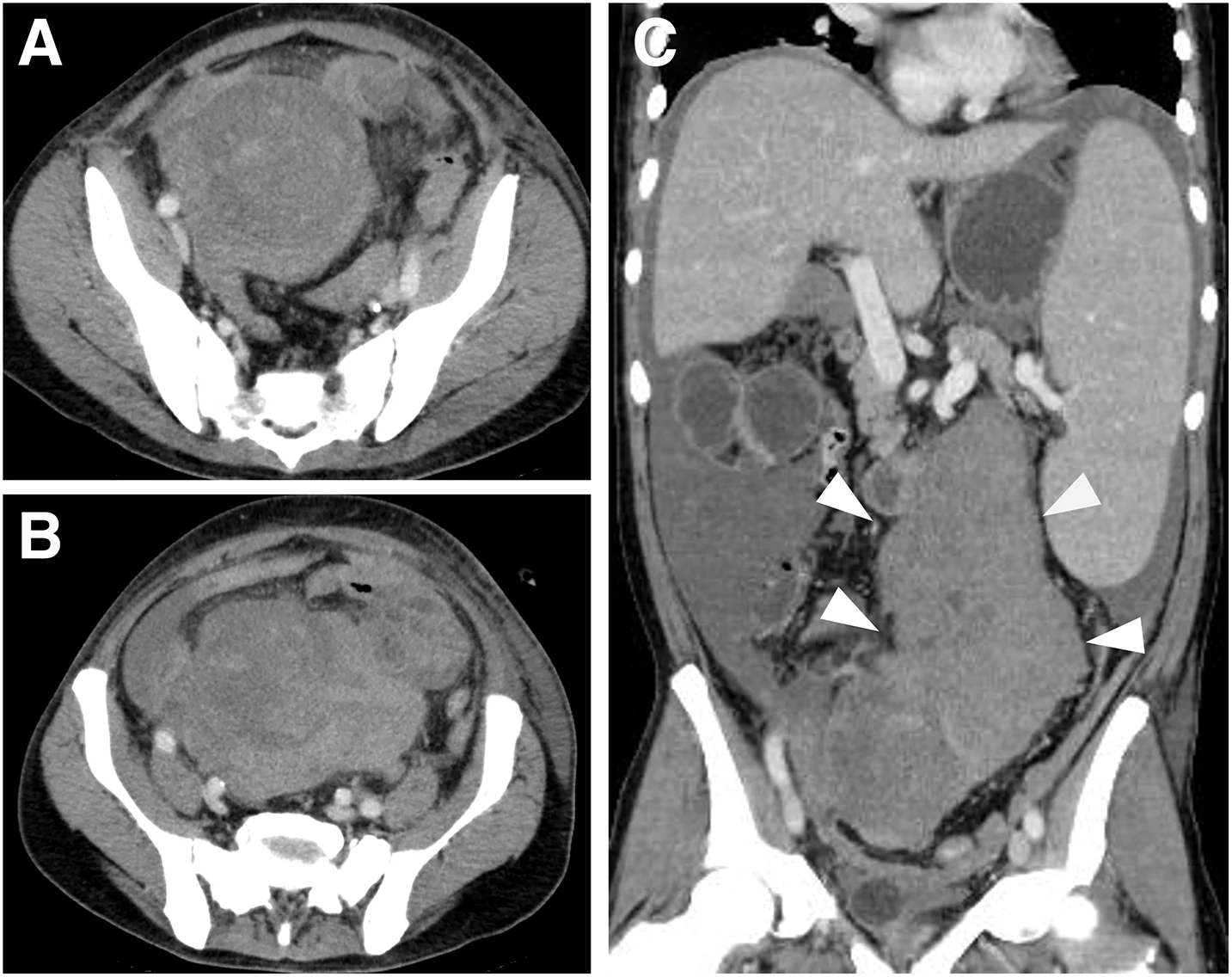
Preoperative contrast-enhanced CT findings. (**A**) Axial image showing a 9-cm pelvic mass with poor enhancement. (**B**) Axial image demonstrating focal wall disruption of the pelvic mass with surrounding hyperdense hematoma. (**C**) Coronal image indicating rupture of the pelvic mass, surrounded by hyperdense hematoma (arrowheads) and hemoperitoneum.

A 62-year-old man with a history of ET presented with acute abdominal pain and was referred by the hematology department of Japan Agricultural Co-operatives Toride Medical Center. Contrast-enhanced CT revealed a 9-cm pelvic mass with poor enhancement and surrounding high-attenuation fluid, indicating its rupture (**Fig. 1**).

**Fig. 2:**
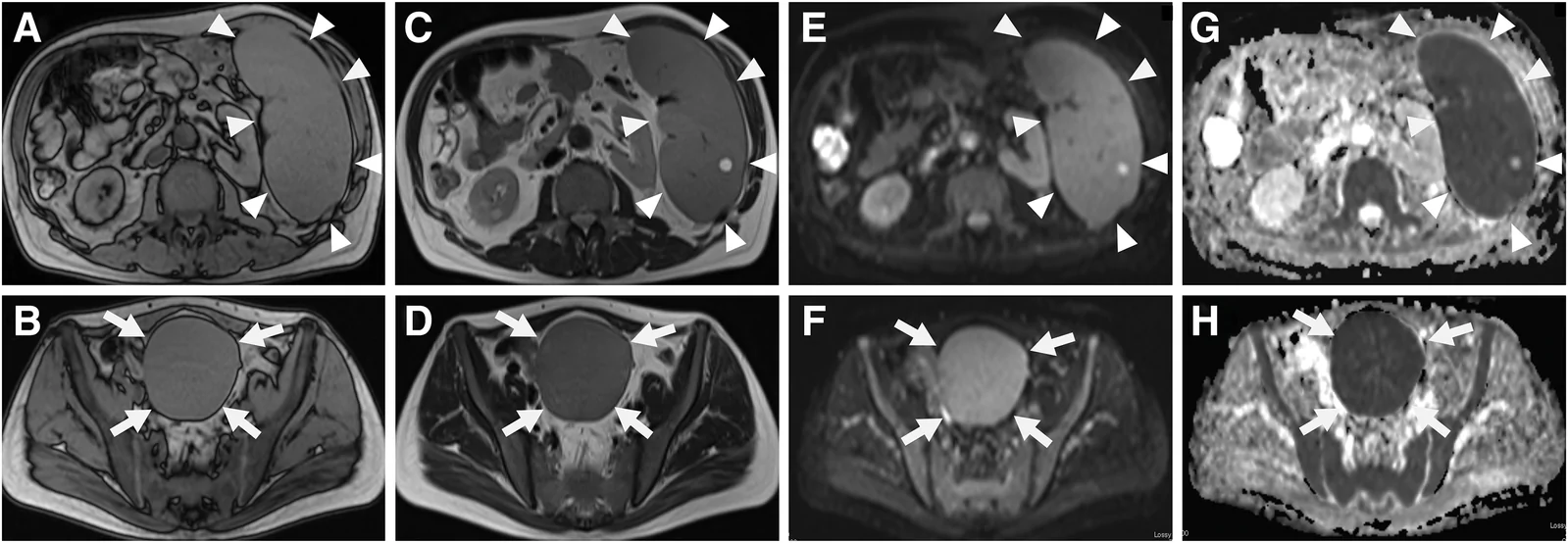
MRI findings at X–2 months. Each panel showed the spleen (upper row) and the pelvic mass (lower row), which demonstrated identical signal characteristics across all sequences (isointense on T1-weighted images, slightly hypointense on T2-weighted images, hyperintense on DWI, and low ADC values). (**A**) Axial T1-weighted image of the spleen (arrowheads). (**B**) Axial T1-weighted image of the pelvic mass (arrows). (**C**) Axial T2-weighted image of the spleen (arrowheads). (**D**) Axial T2-weighted image of the pelvic mass (arrows). (**E**) Axial DWI of the spleen (arrowheads). (**F**) Axial DWI of the pelvic mass (arrows). (G) Axial ADC map of the spleen (arrowheads). (**H**) Axial ADC map of the pelvic mass (arrows). ADC, apparent diffusion coefficient; DWI, diffusion-weighted imaging

**Fig. 3:**
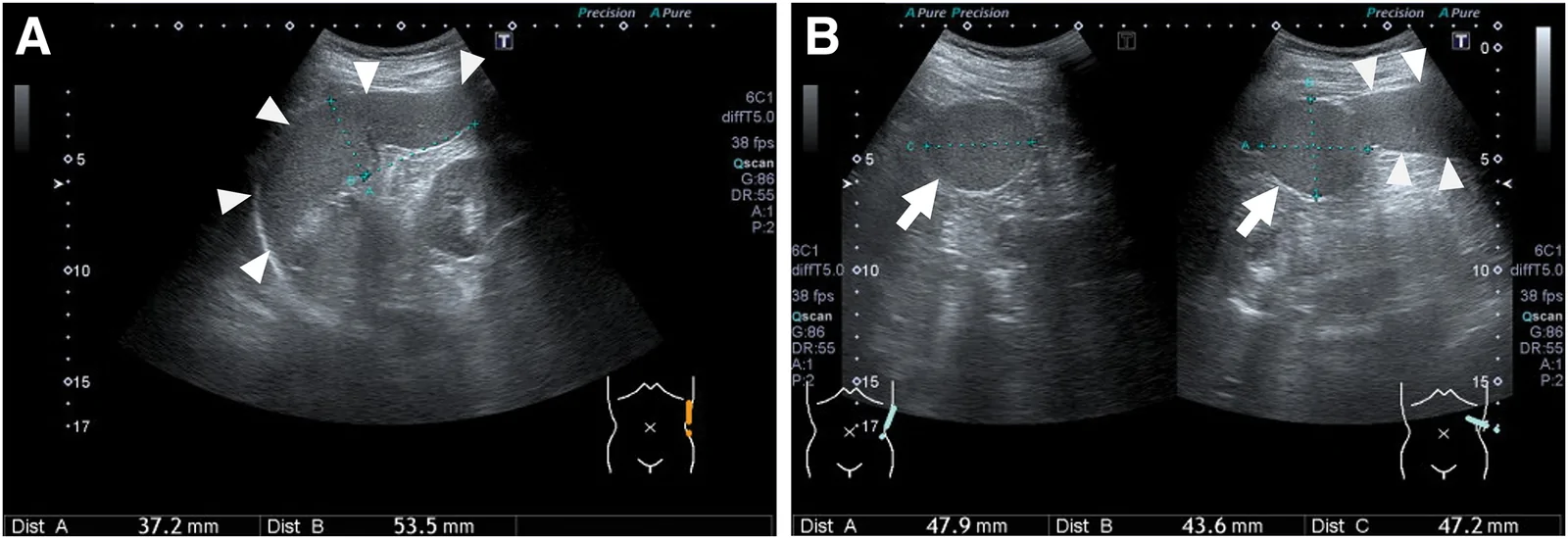
US findings at X–8 years. (**A**) Mild splenomegaly was detected in the left upper abdomen (arrowheads) at the time of the initial diagnosis of ET. (**B**) A 4.8-cm well-defined round mass was detected (arrow) adjacent to the native spleen (arrowheads). The mass was considered as an accessory spleen. ET, essential thrombocythemia; US, ultrasonography

A routine health checkup conducted 8 years ago revealed an elevated platelet count of approximately 500 × 10^9^/L. The patient was referred to our hospital’s Hematology Department by his family doctor. Genetic testing revealed a JAK2 V617F mutation, and the patient was subsequently diagnosed with ET. He was treated with hydroxyurea, a DNA synthesis inhibitor, and anagrelide, a megakaryocyte proliferation suppressor; however, the disease was uncontrolled and gradually deteriorated. CT revealed splenomegaly and an 8-cm pelvic mass 2 months prior. Splenomegaly was considered a result of secondary myelofibrosis; however, a pelvic mass was not diagnosed. The pelvic mass was round and well-defined, and the feeding vessels arose from the left gastroepiploic vessels. MRI was also performed 2 months prior, and the signal characteristics of the pelvic mass were identical to those of the native spleen on T1- and T2-weighted images, DWI, and ADC maps, suggesting an accessory spleen rather than a neoplasm (**Fig. 2**). The other previous imaging study was an abdominal US performed 8 years prior. This examination demonstrated mild splenomegaly and a well-defined, round 4.8-cm mass adjacent to the native spleen. In retrospect, this mass was considered consistent with an accessory spleen (**Fig. 3**). These findings indicate that both splenomegaly and enlargement of the accessory spleen had already been present 8 years before the current presentation. No other prior imaging studies were available.

**Fig. 4:**
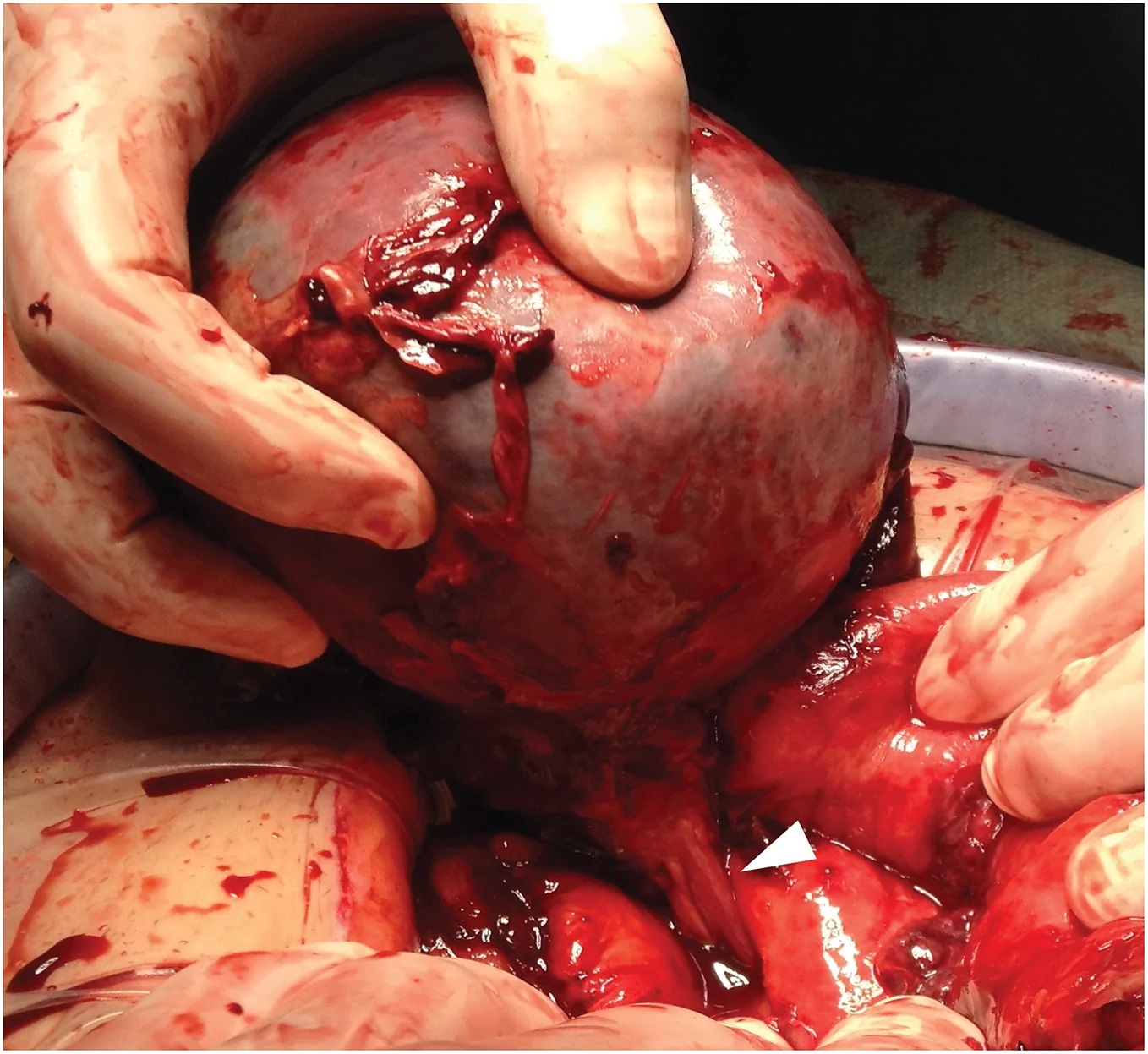
Intraoperative findings. A 9-cm ruptured accessory spleen was only attached to a cord-like vascular pedicle (arrowhead). There was no supportive tissue, except for this vascular pedicle.

His medical history and previous radiological studies were not shared between the hematology department and our department until the onset of the acute abdominal pain; therefore, we had no opportunity to perform prophylactic resection of the wandering pelvic accessory spleen. However, the previously available clinical and imaging information enabled a prompt diagnosis of torsion and rupture of the pelvic accessory spleen at presentation. During the emergency laparotomy, a ruptured 9-cm wandering accessory spleen surrounded by a large hematoma was observed in the pelvis (**Fig. 4**). A cord-like vascular pedicle was the only supporting tissue, and its torsion was identified. The accessory spleen was easily resected by ligating the pedicle. Splenomegaly was observed in the left lateral abdomen.

**Fig. 5:**
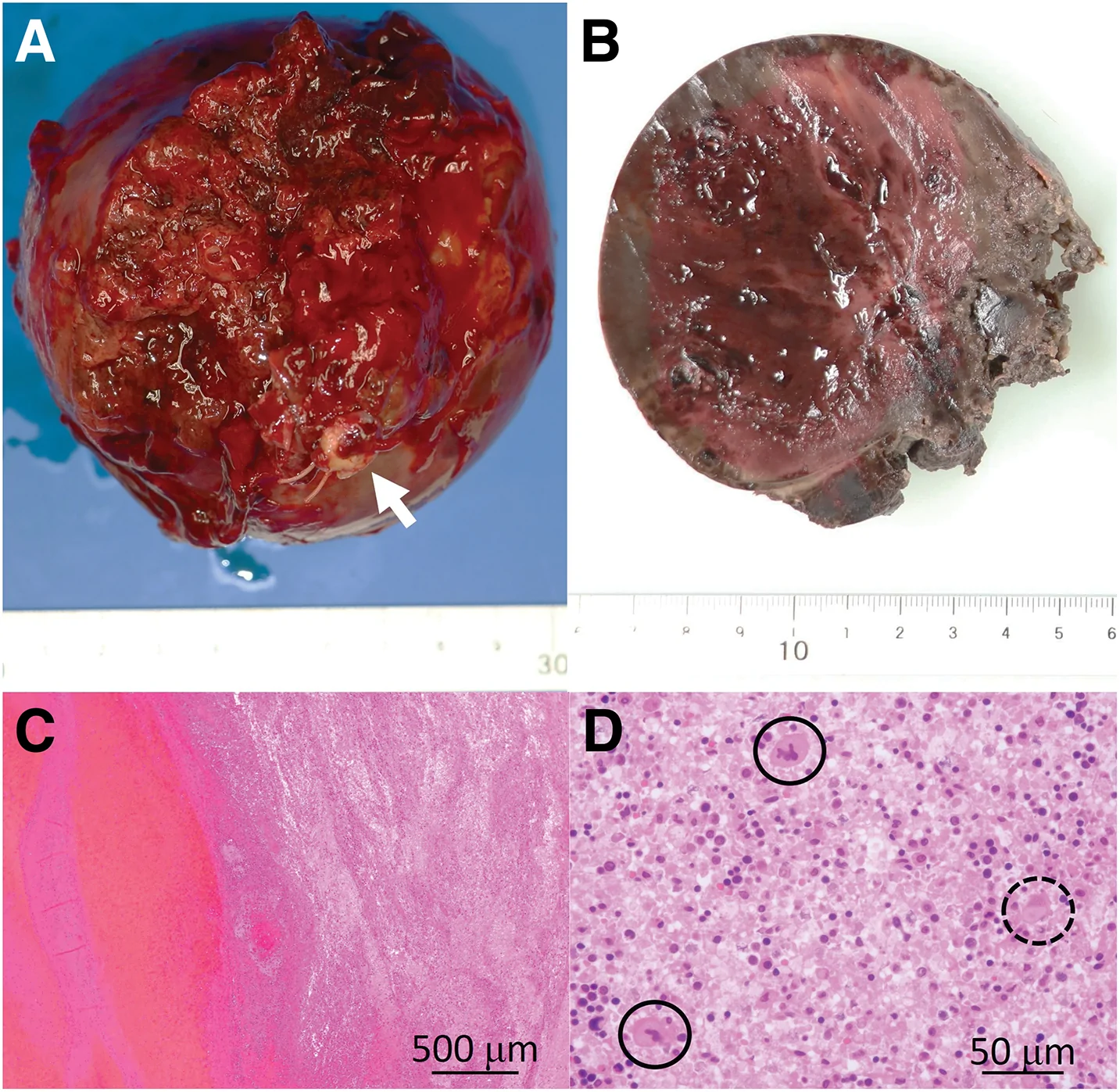
Pathological findings of the resected accessory spleen. (**A**) Macroscopically, the ligated stump of the vascular pedicle was observed (arrow). (**B**) The macroscopic cut surface showed a hematoma within the accessory spleen. (**C**) Microscopically, approximately 90% of the field consisted of necrotic tissue. (**D**) Microscopically, megakaryocytes (circles) and a necrotic megakaryocyte (dotted circle) were identified in the non-necrotic area, indicating extramedullary hematopoiesis in the accessory spleen.

Histopathological examination of the resected accessory spleen revealed extensive necrosis, whereas the non-necrotic area contained megakaryocytes, indicating extramedullary hematopoiesis (**Fig. 5**).

The postoperative course was uneventful, and the patient was discharged on POD 16. After discharge, his treatment was changed to the JAK2 inhibitor, ruxolitinib. At the 1-year follow-up, his general condition remained good, with platelet counts maintained at approximately 200 × 10^9^/L.

## DISCUSSION

Accessory spleens are ectopic splenic tissues frequently identified in clinical practice.^1)^ Most accessory spleens are asymptomatic^3)^; however, torsion may occur, leading to acute abdominal pain. This pain occurs mostly in children and young adults.^27)^ The event is relatively rare in older patients, with only 3 cases previously reported in patients aged 60 and over, including this case.^18,28,29)^

The risk of torsion is high in large^4)^ and wandering accessory spleens.^14)^ A wandering accessory spleen has few supportive connective ligaments or lacks supportive tissue, except for an elongated vascular pedicle.^5,21)^ The vascular pedicle forms a cord-like tissue, and its torsion subsequently leads to venous congestion, ischemia, and infarction of the accessory spleen, eventually resulting in hemorrhage or rupture.^4)^

Pelvic accessory spleens have been previously reported in 18 cases, including this case (**Table 1**).^6–22)^ Torsion occurred in 50% of the cases. Overall, 13 (72%) of the 18 reported cases of pelvic accessory spleen were described as wandering accessory spleen. It is difficult to diagnose a pelvic accessory spleen preoperatively. Most patients were diagnosed intraoperatively or by postoperative pathological examination (67%). Other differential diagnoses of a pelvic mass are rectal GIST, omental GIST, uterine adnexal mass, malignant lymphoma, and splenosis.^21,22)^

**Table 1:** Eighteen cases of pelvic accessory spleen

No.	Reference	Year	Age	Sex	Preoperative diagnosis	Size (cm)	Torsion	Vascular pedicle	Past medical history
1	^6)^	1987	38	M	Unknown	6.0	N/A	N/A	N/A
2	^7)^	1993	44	F	Left ovarian tumor	7.0	N/A	Yes	N/A
3	^8)^	1993	N/A	N/A	N/A	N/A	N/A	N/A	N/A
4	^9)^	1999	26	F	Accessory spleen, adnexal mass	4.5	Yes	Yes	N/A
5	^10)^	2001	17	F	Right ovarian tumor	13.0	Yes	Yes	None
6	^11)^	2001	58	F	Accessory spleen	4.0	N/A	Yes	N/A
7	^12)^	2001	26	F	N/A	N/A	Yes	N/A	Splenectomy due to trauma
8	^13)^	2007	18	M	Accessory spleen	5.0	N/A	Yes	N/A
9	^14)^	2014	17	F	Accessory spleen	6.0	No	Yes	None
10	^15)^	2015	43	F	Ovarian tumor	5.5	No	No	Uterine myoma
11	^16)^	2015	38	F	Pelvic neoplasms	3.1 (multiple)	No	No	Splenectomy due to trauma
12	^17)^	2015	67	F	Accessory spleen	3.0	No	Yes	None
13	^18)^	2016	60	M	GIST, malignant lymphoma in the rectum	4.7	No	Yes	HCV, cirrhosis
14	^19)^	2017	N/A	N/A	Adnexal mass	N/A	Yes	N/A	N/A
15	^20)^	2017	18	M	Benign abdominal tumors	6.8	Yes	Yes	None
16	^21)^	2018	39	F	Pelvic neoplasm, GIST	8.0 (multiple)	No	Yes	None
17	^22)^	2020	51	M	Accessory spleen	6.7	No	Yes	None
18	Our Case	2026	62	M	Accessory spleen	9.0	Yes	Yes	ET

ET, essential thrombocythemia; F, female; GIST, gastrointestinal stromal tumor; HCV, hepatitis C virus; M, male; N/A, not available

Useful diagnostic modalities for accessory spleens include US, CT, MRI, and nuclear medicine studies, including Tc-99m SC and Tc-99m HDRBC scintigraphy.^30)^ Accessory spleens are detected as well-defined, homogeneous, round masses using all modalities. Dynamic CT typically shows the same enhancement patterns as the native spleen in the arterial, portal venous, and delayed phases. However, in torsion cases, hemodynamic changes occur; therefore, the enhancement patterns of accessory spleens differ from those of the native spleen. The native and accessory spleens show a similar signal pattern in all MRI sequences.^15,30)^ In this case, the native and accessory spleens were isointense on T1-weighted images, slightly hypointense on T2-weighted images, hyperintense on DWI, and low ADC values. In wandering accessory spleen cases, Doppler US, CT angiography, and MRI are useful for detecting the vascular pedicle.^17,18)^ Nuclear medicine studies such as Tc-99m SC and Tc-99m HDRBC specifically accumulate in the splenic tissue. Tc-99m HDRBC is the most useful modality for identifying accessory spleens.^30)^ PET-CT is another useful diagnostic modality.^18)^ Abnormal accumulation of ^18^F-fluorodeoxyglucose is not observed in accessory spleens but in most malignant tumors.

ET is a myeloproliferative neoplasm characterized by clonal hematopoietic stem cell abnormalities, marked thrombocytosis, megakaryocytic hyperplasia, and symptoms, including bleeding and thrombosis. Approximately 12% of cases progress to secondary myelofibrosis.^23)^ Extramedullary hematopoiesis occurs in the spleen, resulting in splenomegaly.^25,26)^ In this case, both splenomegaly and accessory spleen enlargement were already evident at the time of the initial diagnosis of ET. As the disease progressed, both the native spleen and the accessory spleen increased in size. Comparison with prior ultrasonographic findings suggested that the accessory spleen had gradually migrated from the vicinity of the native spleen to the pelvic cavity over an 8-year period. Simultaneous splenomegaly and accessory splenic enlargement are rare. To the best of our knowledge, this is the first case report describing this pathophysiology in association with hematologic disease. Other hematopoietic diseases, including leukemia and polycythemia vera, can also cause myelofibrosis, extramedullary hematopoiesis, and splenomegaly.^23)^ Patients with these diseases may also be at risk of developing large accessory spleens; however, our literature review identified no previously reported cases describing simultaneous enlargement of both native and accessory spleens in patients with these hematopoietic diseases.

Simultaneous enlargement of both native and accessory spleens has been previously reported in a male patient with cirrhosis associated with HCV.^18)^ In this case, mild splenomegaly and a mildly enlarged wandering pelvic accessory spleen (4.5 cm) were observed. The feeding vessel of the pelvic accessory spleen arises from the gastroepiploic artery and vein. The hemodynamics of the pelvic accessory spleen may be similar to those of the native spleen. These spleens were enlarged owing to portal hypertension associated with cirrhosis.

Conversely, asynchronous compensatory enlargement of the accessory spleen after splenectomy has been frequently reported in patients with AIHA and ITP.^31,32)^ Asynchronous compensatory enlargement of a remnant accessory spleen can lead to recurrence after splenectomy and remains a significant clinical issue in these cases.^3,32)^

Patients with ET are at an increased risk of thrombosis; therefore, careful perioperative anticoagulation management is essential.^24)^ Patients with splenomegaly have a higher risk of thrombosis than those without splenomegaly.^24)^ No standardized perioperative management has been established; however, Tefferi et al. recommended using low-molecular-weight heparin and maintaining perioperative platelet counts below 450 × 10^9^/L.^23)^ In this case, no anticoagulants were administered to prevent potential postoperative bleeding. The postoperative course of the patient was uneventful.

Awareness of the pelvic accessory spleen and its torsion is important for the differential diagnosis of pelvic masses and acute abdominal pain. In the present case, prophylactic resection of the pelvic accessory spleen was not considered because the patient’s clinical history and prior imaging findings had not been shared between the hematology department and our department. Effective sharing of clinical information among surgeons, physicians, and radiologists may facilitate the identification of accessory spleens at risk of torsion and provide an opportunity for prophylactic intervention.

## CONCLUSIONS

We report a rare case of torsion and rupture of a wandering pelvic accessory spleen associated with ET. Awareness of the pelvic accessory spleen and its torsion is important for the differential diagnosis of pelvic masses and acute abdominal pain. Elective resection should be considered to prevent torsion or rupture when a large pelvic accessory spleen is diagnosed. Simultaneous enlargement of both native and accessory spleens is exceptionally rare. Further accumulation of case reports describing this pathophysiology is warranted.

## DECLARATIONS

### Availability of data and materials

The data that support the findings of this study are available from the corresponding author upon reasonable request.
